# Accuracy of the Intra- and Extra-Oral Scanning Technique for Transferring the Intaglio Surface of a Pontic of Provisional Restorations to Definitive Restorations

**DOI:** 10.3390/ma14216489

**Published:** 2021-10-29

**Authors:** Koma Sanda, Noriyuki Yasunami, Maki Okada, Akihiro Furuhashi, Yasunori Ayukawa

**Affiliations:** 1 Section of Implant & Rehabilitative Dentistry, Division of Oral Rehabilitation, Faculty of Dental Science, Kyushu University, Fukuoka 812-8582, Japan; ko-ma@dent.kyushu-u.ac.jp (K.S.); furuhasi@dent.kyushu-u.ac.jp (A.F.); ayukawa@dent.kyushu-u.ac.jp (Y.A.); 2 Department of Medical Technology, Kyushu University Hospital, Fukuoka 812-8582, Japan; okada.maki.518@m.kyushu-u.ac.jp

**Keywords:** accuracy, digital dentistry, digital workflow, fixed partial denture, intraoral scanner, intra-and extra-oral scanning technique, pontic, precision, trueness

## Abstract

When taking the final impression for a three-unit fixed partial denture (FPD), the intaglio surface of the pontic of provisional restoration cannot be transferred accurately to that of definitive restoration. The intra- and extra-oral scanning (IEOS) technique, a method for accurately reproducing the submucosal morphology of the superstructure of an implant, has been reported using an intraoral scanner. In the present study, we evaluated the difference between the conventional impression method using impression material and the IEOS technique in reproducing the morphology of the surface of the pontic of a definitive FPD. There was a significant difference in the trueness of the intaglio surface morphology of the pontic between the conventional method and the IEOS technique; however, no significant difference in precision was observed. As a result, the intaglio surface of the pontic of the three-unit FPD could be transferred to definitive restorations more accurately with the IEOS technique than with the conventional method. These results suggest that the IEOS technique can duplicate the intaglio surface of the pontic more reproducibly to the definitive restorations compared with the conventional method.

## 1. Introduction

The development of the intraoral scanner (IOS) has led to its use gradually spreading [1], because, compared with the conventional method using impression material and gypsum, taking impressions using IOS has many advantages, such as shortened chair time and reduced patient discomfort and risks, such as the aspiration of impression material, reduced deformation caused by the material, the fact that the results are savable as data, and the possibility of simplified data transfer to dental laboratories [2,3,4,5].

The comparison of dimensional accuracy of digital methods generated by intraoral scanning or conventional methods using alginate or silicone has been reported in several studies [6,7,8]. Most studies reported that intraoral scanners are either more accurate or as good as conventional materials [6,7,9]. When the region of interest increases while using the digital impression technique, trueness and accuracy tend to decrease [10]. However, when scanned under a three-unit area, more accuracy was reported compared to conventional methods [11].

In fixed partial denture (FPD) treatment, the intaglio surface of the pontic should be considered with respect to cleanability [12]. The esthetics, including the intaglio surface of the pontic and soft tissue shape, are also important [13]. While the fixed partial denture is fabricated in the esthetic zone, provisional restoration with the ovate pontic is usually utilized to form the esthetic gingival profile. The provisional pontic is often modified to produce an esthetic and harmonious gingival profile; then, a definitive impression is taken.

However, while taking definitive impressions, the mucosal morphology compressed by the provisional pontic immediately changes after the removal of provisional restorations [14]. The conventional method requires several minutes to set the impression material, and the mucosal morphology may change during this time. As a result, the gingival profile generated by the surface of the provisional pontic cannot be transferred accurately to a working cast [14]. In contrast, when impressions are taken using IOS, the mucosal morphology can be taken immediately after the removal of the provisional restorations to minimize soft tissue changes [14]. However, even when using this approach, the intaglio surface of the pontic cannot be accurately transferred from provisional restorations to definitive restorations, because the gingival morphology may change immediately after the removal of the provisional restoration [14].

The intra- and extra-oral scanning (IEOS) technique, used as a method for accurately reproducing the submucosal morphology for the superstructure of the implant, has been reported using IOS [15]. The IEOS technique is thought to be applied not only to the reproduction of the subgingival contour of the implant’s definitive restoration but also to the reproduction of the intaglio surface of the pontic in the natural-tooth FPD. To the best of our knowledge, there has been no report of making a definitive restoration (FPD of a natural tooth) using the IEOS technique or investigation of the accuracy (trueness and precision) of the intaglio surface of the pontic.

The purpose of this study was to compare the morphological accuracy (trueness and precision) of the intaglio surface of the pontic between provisional and definitive restorations produced with the conventional method or IEOS technique in FPDs of natural teeth. This in vitro assessment of the IEOS technique is important in preclinical testing to further investigate the usefulness of utilizing the IEOS technique for patients in the future. The null hypothesis of this study is that there is no difference in the accuracy of the intaglio surface of the pontic of the definitive restorations prepared by the conventional method and the IEOS technique.

## 2. Materials and Methods

**Figure 1:**
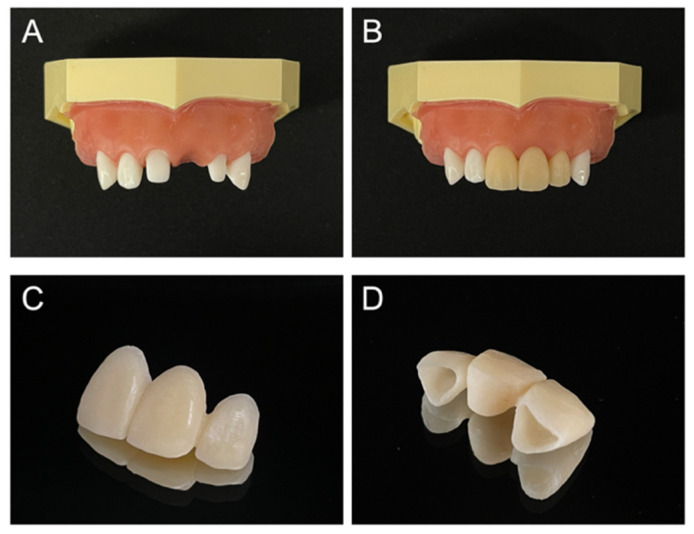
(**A**) Tooth preparation-formed model. (**B**) Provisional restoration attached. (**C**,**D**) Provisional restoration (ovate pontic).

A typodont with artificial gingiva attached (D16-500HPRO-S1A1-GSE, NISSIN, Kyoto, Japan) was used. The artificial teeth (A55A-111, A55A-221, NISSIN) with a margin of deep chamfer were attached at positions 11 (the maxillary right central incisor) and 22 (the maxillary left lateral incisor). The 21 (the maxillary left central incisor) position was partially edentulous, and the gingiva was mimicked with silicone rubber (Figure 1A). Provisional restoration of the three-unit FPD was produced and attached to this typodont (Figure 1B). The provisional restoration was waxed-up, and the morphology was obtained using laboratory putty (Blue eco, Detax, Ettlingen, Germany) and prepared by using an immediate polymerization resin (PROVINICE, Shofu, Kyoto, Japan). The pontic basal plane was of the ovate type, and it was adjusted so that it pressed the mucosal surface (Figure 1C,D).

### 2.1. Conventional Impression Method

**Figure 2:**
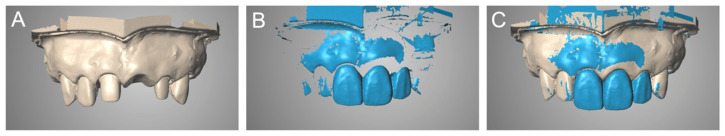
Scan data of cast created by the conventional method. (**A**) Working casts. (**B**) Form reference cast of the provisional restoration. (**C**) The two STL data were superposed.

**Figure 3:**
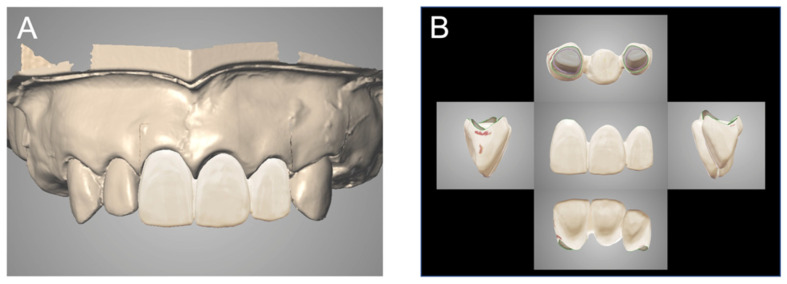
The digital wax-up of definitive restoration was completed by the conventional method. (**A**) Provisional restoration attached. (**B**) Provisional restoration.

For the conventional method, impressions were taken with silicone impression material (Panasil, Kettenbach Dental, Eschenburg, Germany) by using an individual tray (Tray Resin II, Shofu). The working cast was made with gypsum (New Fujirock, GC, Tokyo, Japan) (Model 1). The form reference casts of the provisional restorations were made with dental stone (New Plastone II, GC) (Model 2). Thereafter, the two casts (Models 1, 2) were scanned with a lab scanner (CARES D7 Plus, Straumann, Basel, Switzerland) (Figure 2). The definitive restorations were designed with computer-aided design software (CARES Visual, Straumann) and then produced by computer-aided manufacturing (Figure 3). The process up to this point was repeated five times. Five digital wax-ups were made using the conventional method and saved as stereolithography (STL) data (sample size: n = 5). The CAD design process steps were designed by a single user, who was an experienced dental technician (M.O.).

### 2.2. The IEOS Technique

**Figure 4:**
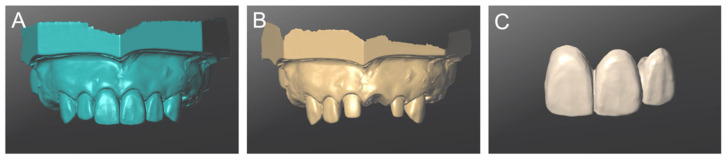
Scan data of cast created by the intra- and extra-oral scanning (IEOS) technique. (**A**) Provisional restoration attached (Data 1). (**B**) Tooth preparation formed model (Data 2). (**C**) Provisional restoration (Data 3).

The workflow for fabricating definitive restoration using the IEOS technique was performed in accordance with the previous report [15]. First, the provisional restoration was attached to the typodont (abutment tooth: 11, 22) and scanned (Data 1) (Figure 4A). Subsequently, the typodont was scanned with the provisional restoration removed (Data 2) (Figure 4B). Finally, the provisional restoration alone was scanned (Data 3) (Figure 4C).

**Figure 5:**
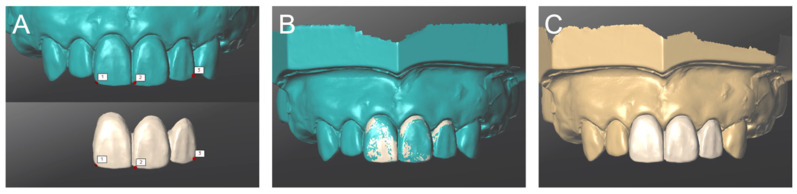
(**A**) After determining three points as reference points, Data 1 (Figure 4A) and Data 3 (Figure 4C) were superimposed. (**B**) Two data (Data 1,3) were superposed. (**C**) Finally, Data 2 (Figure 4B) was superposed.

**Figure 6:**
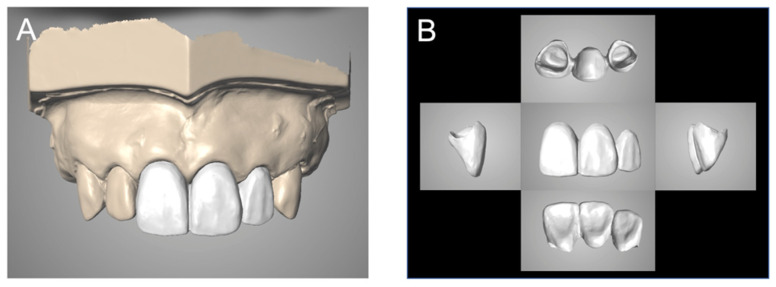
The digital wax-up of definitive restoration was completed by the intra- and extra-oral scanning (IEOS) technique. (**A**) Provisional restoration attached. (**B**) Provisional restoration.

The three STL data files (Data 1–3) were imported and superimposed in computer-aided design software (CARES Visual) (Figure 5). The completed digital wax-up was saved as STL data (Figure 6). The process up to this point was repeated five times. Five digital wax-ups were produced using the IEOS technique and saved as STL data (sample size: n = 5). The CAD design process steps were designed by a single user, who was an experienced dental technician (M.O.). When using IOS, it was carried out in an indoor environment where sunlight was blocked and fluorescent lights were on. A trained researcher (K.S.) performed all the scans in accordance with each manufacturer’s instructions.

To compare the resulting STL data, the intaglio surface of the pontic of definitive and provisional restorations was measured with morphometry software (PolyWorks Inspector, InnovMetric Software, Quebec City, QC, Canada).

### 2.3. Measurement

**Figure 7:**
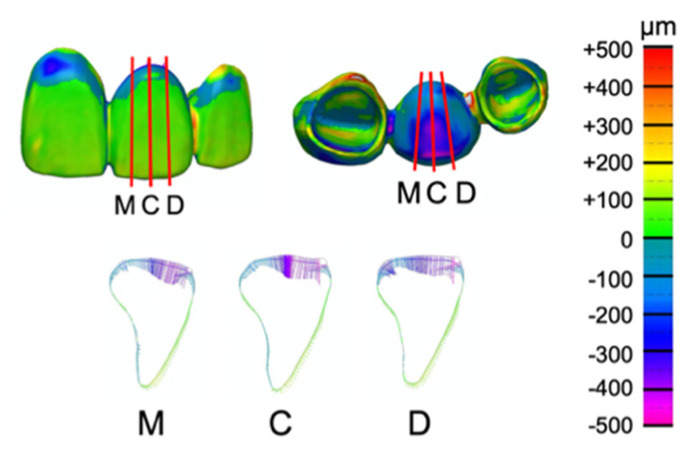
Created “cross-sections” with morphometry software. Mesial cross-section (M). Central cross-section (C). Distal cross-section (D).

In the evaluation of trueness, provisional restoration as reference data and STL data of definitive restorations were superimposed using morphometry software (comparison with reference data: n = 5). In the evaluation of precision, STL data of the definitive restorations were superposed on the morphometry software (comparison within the group: n = 10). With reference to the research so far, the “best-fit method” was used for superimposition [16,17,18,19]. The distortions between superimposed STL layers were then measured by using the “cross-section” function of the software. The three cross-sections were made quarterly, based on the mesiodistal width of the pontic. The three cross-sections selected were mesial, central, and distal; the largest distortion was recorded in each cross-section (Figure 7). The mean value of distortion in each cross-section was measured.

### 2.4. Statistical Analysis

Statistical analyses were performed to compare the obtained data. The Kolmogorov–Smirnov test was performed to test data for normality. Since normality was confirmed, a parametric test (F-test) was performed. As a result, it was homoscedastic, so the Student’s *t*-test analysis was performed. All statistical analyses were performed with EZR software, which is an interface for R (R version 3.6.3, The R Foundation, Vienna, Austria) [20]. Differences were considered significant if *p*-values were < 0.05.

## 3. Results

### 3.1. Trueness

**Figure 8:**
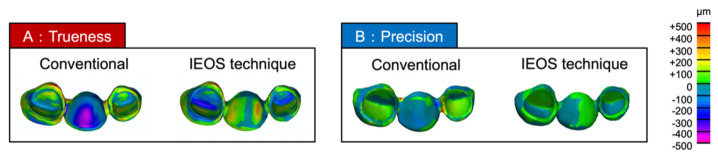
(**A**) Trueness: STL data of provisional and definitive restorations were superimposed by software. (**B**) Precision: STL data of each definitive restoration were superimposed on the software.

**Figure 9:**
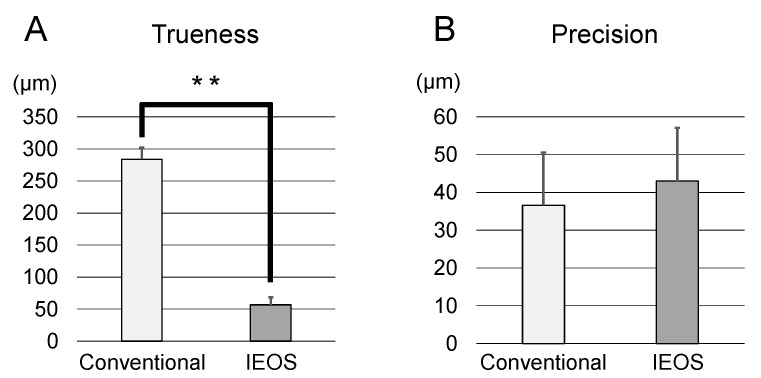
Results of statistical analysis (Student’s *t*-test, **: *p* < 0.01). (**A**) Trueness. (**B**) Precision.

In the measurement of trueness, the intaglio surface of the pontic of definitive restorations made with the conventional impression method showed greater distortion compared with those made with the IEOS technique (Figure 8A). The intaglio surface of the pontic of the definitive restoration fabricated by the conventional method exhibited a trueness of 283.9 ± 19 μm (range from 253.4–300.2 μm) compared with the provisional restoration (Table 1). The intaglio surface of the pontic of the definitive restoration produced by the IEOS technique exhibited a trueness of 56.6 ± 12 μm (range from 49.1–77.3 μm) compared with the provisional restoration (Table 1). As a result of statistical analysis, the trueness of the intaglio surface of the pontic of the definitive restoration produced by the IEOS technique was higher (the morphological difference was smaller) than that of the conventional method with statistical significance (*p* < 0.001) (Table 1, Figure 9A).

### 3.2. Precision

For the measurement of precision, in the comparison with the intaglio surface of the pontic of the definitive restoration prepared by the conventional method, the morphology was almost the same as a whole (there was almost no difference in morphology) (Figure 8B). The intaglio surface of the pontic of the definitive restoration produced by the conventional method exhibited a precision of 36.6 ± 14 μm (range from 17.5–56.7 μm) compared with the provisional restoration (Table 1). The intaglio surface of the pontic of the definitive restoration produced by the IEOS technique exhibited a precision of 43.0 ± 14 μm (range from 23.6–66.2 μm) compared with the provisional restoration (Table 1). As a result of statistical analysis, no significant difference was found in the precision of the intaglio surface of the pontic of the definitive restoration prepared by the conventional method and the IEOS technique (*p* = 0.32) (Table 1, Figure 9B).

## 4. Discussion

The null hypothesis of the present study was that “There is no difference in accuracy (trueness and precision) in the intaglio surface of the pontic of the definitive restorations prepared by the conventional method and the IEOS technique”. To investigate this hypothesis, we compared the definitive restorations produced with the conventional method and the IEOS technique in morphometry software. The trueness of the intaglio surface of the pontic of the definitive restoration produced by the IEOS technique was significantly higher than that produced by the conventional method. On the other hand, there was no significant difference in the precision. Therefore, the null hypothesis was partially rejected.

Esthetic parameters are focused on not only prosthesis itself (white esthetic score) but also on soft tissue around the teeth (pink esthetic score) [21,22]. In this context, in addition to the soft tissue grafting techniques, the subgingival contour of the prosthesis, which supports soft tissue, is one of the essential factors needed to achieve an esthetic outcome [23,24].

In the present study, the intaglio surface of the pontic of definitive restorations produced with the conventional impression method showed greater distortion compared with the restorations produced with the IEOS technique. When the provisional restoration is removed while taking the impression with the conventional method, distress induces the deformation of the mucosal morphology under the pontic. This may cause inaccuracy of the gingival morphology on the working cast, which may cause a misfit between the definitive pontic and mucosa. That is, the morphology of the provisional pontic cannot be reproduced accurately with the definitive pontic using the conventional method [14]. On the other hand, when impressions are taken by IOS, scanning (impression taking) can be performed immediately after the removal of the provisional restorations to minimize mucosa changes [19]. However, even with this method, mucosal morphology deformation has been reported [14]. Therefore, we tested the IEOS technique [15] as a method to solve this problem in the present study. By using the IEOS technique, regardless of whether the mucosal morphology deforms after the removal of provisional restorations, the intaglio surface of the pontic could be transferred from provisional restorations to definitive restorations.

In the conventional method, distortion may occur in several steps while fabricating the working cast. In impression taking, using elastic material such as silicone rubber, the impression material is deformed during the impression tray removal [25]. Furthermore, when producing the working cast with gypsum, deformation due to hygroscopic expansion during setting also occurs [26]. The use of IOS can eliminate the deformation during the fabrication of gypsum working casts [27,28,29]. This is in agreement with the results of the present study. Our results suggested that the IEOS technique can transfer the intaglio surface of the pontic to definitive restoration more accurately than the conventional method.

In dental treatment, it is necessary to reproduce the correct shape of the teeth onto the working cast. Clinically acceptable deviation from study models is reported to be 300 μm in pedodontics [30] and 200 μm in orthodontics [31]. In prosthodontic treatment, it has been reported that the permissible range of impression precision differs depending on the type of prosthesis, but the permissible range of a single crown is between 0 and 120 μm [32]. The precision results in the present study meet this criterion for both the conventional method (36.6 ± 14 μm: range from 17.5 to 56.7 μm) and the IEOS technique (43.0 ± 14 μm: range from 23.6–66.2 μm). In addition, the precision of digital impression taking by means of an IOS is likely influenced by a number of factors, including the operator’s proficiency, the performance of IOS, the scan path procedure, and the scanning environment [33,34,35]. Therefore, all digital impressions were performed by the manufacturer’s recommended scanning methods (dry state, no shadowless light, no scan powder, appropriate scan path, etc.). Thus, the result was that “There was no significant difference in the precision of the intaglio surface of the pontic of the definitive restoration prepared by the conventional method and the IEOS method.” Therefore, it was suggested that the IEOS technique could produce definitive restoration with the same precision as the conventional method and may be clinically applicable.

Finally, this study had several limitations. First, this study was an experiment using typodont with silicone gingiva, and the degree of mucosal deformation due to compression is different from that of actual gingiva in clinical practice. In clinical practice, it has been reported that the accuracy of IOS was reduced by impaired factors such as saliva, and the same results as those in this experiment are not always obtained. Second, the definitive restorations were not milled, but STL data were compared. The reason for this was to reduce the accuracy error due to the CAM machine. After digital wax-up, the definitive restoration was milled by the CAM machine in a clinical situation. At that time, additional errors may have occurred depending on the model of the CAM machine and the selected material for the definitive prosthesis. Third, this study was carried out only in a specific setting of IOS, which means these data are not universal for any other system. Further research is also needed for these settings.

## 5. Conclusions

In the production of definitive restoration of a natural-tooth FPD, the production method using the IEOS technique was able to transfer the intaglio surface of the pontic more accurately (in trueness) compared with the conventional method. Therefore, it is suggested that the IEOS technique may be a useful method to accurately reproduce the intaglio surface of the pontic of provisional restoration to definitive restoration.

## Figures and Tables

**Table 1:** Mean ± SD values and statistical analysis of accuracy (trueness and precision).

Accuracy	Mean ± SD		*p*-Value
	Conventional Method	IEOS Technique	
Trueness	283.9 ± 19 μm	56.6 ± 12 μm	<0.001 **
Precision	36.6 ± 14 μm	43.0 ± 14 μm	0.32

Differences were considered significant if *p*-values were < 0.05. ** Denotes a significant difference at *p* < 0.01.

## Data Availability

The primary data that support the results described here are available from the corresponding author upon reasonable request.

## References

[1] Mangano F., Gandolfi A., Luongo G., Logozzo S. (2017). Intraoral scanners in dentistry: A review of the current literature. BMC Oral Health.

[2] Koulivand S., Ghodsi S., Siadat H., Alikhasi M. (2020). A clinical comparison of digital and conventional impression techniques regarding finish line locations and impression time. J. Esthet. Restor. Dent..

[3] Del Corso M., Abà G., Vazquez L., Dargaud J., Ehrenfest D.M.D. (2009). Optical three-dimensional scanning acquisition of the position of osseointegrated implants: An in vitro study to determine method accuracy and operational feasibility. Clin. Implant Dent. Relat. Res..

[4] Ahlholm P., Sipilä K., Vallittu P., Jakonen M., Kotiranta U. (2018). Digital versus conventional impressions in fixed prosthodontics: A review. J Prosthodont..

[5] Schepke U., Meijer H.J.A., Kerdijk W., Cune M.S. (2015). Digital versus analog complete-arch impressions for single-unit premolar implant crowns: Operating time and patient preference. J. Prosthet. Dent..

[6] Flügge T.V., Schlager S., Nelson K., Nahles S., Metzger M.C. (2013). Precision of intraoral digital dental impressions with iTero and extraoral digitization with the iTero and a model scanner. Am. J. Orthod. Dentofac. Orthop..

[7] Hayashi K., Sachdeva A.U., Saitoh S., Lee S.-P., Kubota T., Mizoguchi I. (2013). Assessment of the accuracy and reliability of new 3-dimensional scanning devices. Am. J. Orthod. Dentofac. Orthop..

[8] Anh J.-W., Park J.-M., Chun Y.-S., Kim M., Kim M. (2016). A comparison of the precision of three-dimensional images acquired by 2 digital intraoral scanners: Effects of tooth irregularity and scanning direction. Korean J. Orthod..

[9] Wiranto M.G., Engelbrecht W.P., Tutein Nolthenius H.E., van der Meer W.J., Ren Y. (2013). Validity, reliability, and reproduci-bility of linear measurements on digital models obtained from intraoral and cone-beam computed tomography scans of alginate impressions. Am. J. Orthod. Dentofacial. Orthop..

[10] Ender A., Zimmermann M., Mehl A. (2019). Accuracy of complete- and partial-arch impressions of actual intraoral scanning systems in vitro. Int. J. Comput. Dent..

[11] Kamimura E., Tanaka S., Takaba M., Tachi K., Baba K. (2017). In vivo evaluation of inter-operator reproducibility of digital dental and conventional impression techniques. PLoS ONE.

[12] Johnson G.K., Leary J.M. (1992). Pontic design and localized ridge augmentation in fixed partial denture design. Dent. Clin. N. Am..

[13] Orsini G., Murmura G., Artese L., Piattelli A., Piccirilli M., Caputi S. (2006). Tissue healing under provisional restorations with ovate pontics: A pilot human histological study. J. Prosthet. Dent..

[14] Su T.-S., Sun J. (2016). Comparison of marginal and internal fit of 3-unit ceramic fixed dental prostheses made with either a conventional or digital impression. J. Prosthet. Dent..

[15] Sasada Y., Huynh-Ba G., Funakoshi E. (2019). Transferring subgingival contours around implants and the intaglio surface of the pontic to definitive digital casts by using an intraoral scanner: A technique. J. Prosthet. Dent..

[16] Miyoshi K., Tanaka S., Yokoyama S., Sanda M., Baba K. (2020). Effects of different types of intraoral scanners and scanning ranges on the precision of digital implant impressions in edentulous maxilla: An in vitro study. Clin. Oral Implant. Res..

[17] Kim J., Son K., Lee K.-B. (2020). Displacement of scan body during screw tightening: A comparative in vitro study. J. Adv. Prosthodont..

[18] Lim J.-H., Park J.-M., Kim M., Heo S.-J., Myung J.-Y. (2018). Comparison of digital intraoral scanner reproducibility and image trueness considering repetitive experience. J. Prosthet. Dent..

[19] Güth J.-F., Keul C., Stimmelmayr M., Beuer F., Edelhoff D. (2013). Accuracy of digital models obtained by direct and indirect data capturing. Clin. Oral Investig..

[20] Kanda Y. (2013). Investigation of the freely available easy-to-use software ‘EZR’ for medical statistics. Bone Marrow Transpl..

[21] Fürhauser R., Florescu D., Benesch T., Haas R., Mailath G., Watzek G. (2005). Evaluation of soft tissue around single-tooth implant crowns: The pink esthetic score. Clin. Oral Implant. Res..

[22] Belser U.C., Grütter L., Vailati F., Bornstein M.M., Weber H.-P., Buser D. (2009). Outcome evaluation of early placed maxillary anterior single-tooth implants using objective esthetic criteria: A cross-sectional, retrospective study in 45 patients with a 2- to 4-year follow-up using pink and white esthetic scores. J. Periodontol..

[23] Khoury F., Happe A., Hoppe A. (2001). Soft tissue management in oral implantology: A review of surgical techniques for shaping an esthetic and functional peri-implant soft tissue structure. Quintessence Int..

[24] Chee W. (2001). Provisional restorations in soft tissue management around dental implants. Periodontol. 2000.

[25] Chen S., Liang W., Chen F. (2004). Factors affecting the accuracy of elastometric impression materials. J. Dent..

[26] Millstein P. (1992). Determining the accuracy of gypsum casts made from type IV dental stone. J. Oral Rehabil..

[27] Camardella L.T., Breuning H., Vilella O. (2017). Accuracy and reproducibility of measurements on plaster models and digital models created using an intraoral scanner. J. Orofac. Orthop. Fortschr. Kieferorthopädie.

[28] Tomita Y., Uechi J., Konno M., Sasamoto S., Iijima M., Mizoguchi I. (2018). Accuracy of digital models generated by conventional impression/plaster-model methods and intraoral scanning. Dent. Mater. J..

[29] Seelbach P., Brueckel C., Wöstmann B. (2013). Accuracy of digital and conventional impression techniques and workflow. Clin. Oral Investig..

[30] Kaihara Y., Kihara T., Kakayama A., Amano H., Nikawa H., Kozai K. (2013). Accuracy of a non-contact 3D measuring system for dental model analysis. Pediatr. Dent. J..

[31] Bell A., Ayoub A.F., Siebert P. (2003). Assessment of the accuracy of a three-dimensional imaging system for archiving dental study models. J. Orthod..

[32] Nawafleh N.A., Mack F., Evans J.L., Mackay J., Hatamleh M.M. (2013). Accuracy and reliability of methods to measure marginal adaptation of crowns and FDPs: A literature review. J. Prosthodont..

[33] Müller P., Ender A., Joda T., Katsoulis J. (2016). Impact of digital intraoral scan strategies on the impression accuracy using the TRIOS Pod scanner. Quintessence Int..

[34] Arakida T., Kanazawa M., Iwaki M., Suzuki T., Minakuchi S. (2018). Evaluating the influence of ambient light on scanning trueness, precision, and time of intra oral scanner. J. Prosthodont. Res..

[35] Ting-Shu S., Jian S. (2015). Intraoral digital impression technique: A review. J. Prosthodont..

